# A life-threatening, massive subcutaneous hematoma caused by trauma in a patient with neurofibromatosis type 1: a case report and literature review

**DOI:** 10.3389/fonc.2024.1387966

**Published:** 2024-05-31

**Authors:** Lu Zhang, Xingtong Wang, Huinan Yin, Wanli Chu, Ming Zhang, Minhui Zhu, Zhiyuan Shi, Zequn Chen, Fan Zhao

**Affiliations:** Burns and Plastic Surgery Department, The Fourth Medical Center of the General Hospital of the People’s Liberation Army, Beijing, China

**Keywords:** case report, hematoma, trauma, neurofibromatosis 1, literature review

## Abstract

**Background:**

Neurofibromatosis type 1 (NF1) is an autosomal dominant disease that can give rise to the formation of vascular lesions in affected individuals. These lesions, whether occurring spontaneously or as a result of trauma, have the potential to cause severe and even fatal hemorrhage.

**Case description:**

We presented a case demonstrating the most extensive hematoma ever documented in a patient with NF1, resulting from a minor trauma. He experienced hemodynamic instability due to severe anemia. Arteriography revealed a rupture in the intercostal artery, which was successfully treated through interventional embolization to stop the hemorrhage. Additionally, we implemented a refined surgical approach, beginning with suturing, followed by the meticulous resection of necrotic and aberrant tissues, thereby markedly diminishing bleeding.

**Conclusion:**

Minor trauma may cause severe bleeding in patients with NF1, which can be life-threatening. Timely diagnosis of NF1 and effective hemostatic techniques are key to successful treatment.

## Highlights

- We presented a case demonstrating the most extensive hematoma ever documented in a patient with NF1, resulting from a minor trauma.
- Endovascular embolization effectively and swiftly halted bleeding, restoring hemodynamic stability.
- A refined surgical approach, beginning with suturing, followed by the resection of necrotic and aberrant tissues, markedly diminished intraoperative bleeding.

## Introduction

Neurofibromatosis type 1 (NF1), known as von Recklinghausen’s disease, is an autosomal dominant disease caused by mutations in the NF1 gene encoding neurofibromin on chromosome 17q11.2 (1). The incidence rate is approximately 1 in every 2500–3000 people worldwide, irrespective of sex or ethnic origin (2). It usually affects the skin, nervous system, and bones and can also cause vascular lesions in patients (3). The prevalence of vascular lesions associated with NF1 ranges between 0.4% and 6.4% (4). Given that numerous vascular lesions associated with NF1 are asymptomatic, the true incidence rate may be greater than currently estimated (5). Vascular lesions include increased vascular fragility, stenosis, aneurysms, pseudoaneurysms, and arteriovenous malformations (6). Spontaneous or traumatic vascular rupture in patients of this kind can lead to life-threatening hemorrhage (7). In this case study, we detailed the progression of a patient with NF1 who, after a minor traumatic injury on the trunk, experienced a severe and potentially fatal subcutaneous hemorrhage. This is the largest subcutaneous hematoma case reported so far, caused by diffuse cutaneous neurofibromatosis originating from the trunk.

## Case presentation

**Figure 1:**
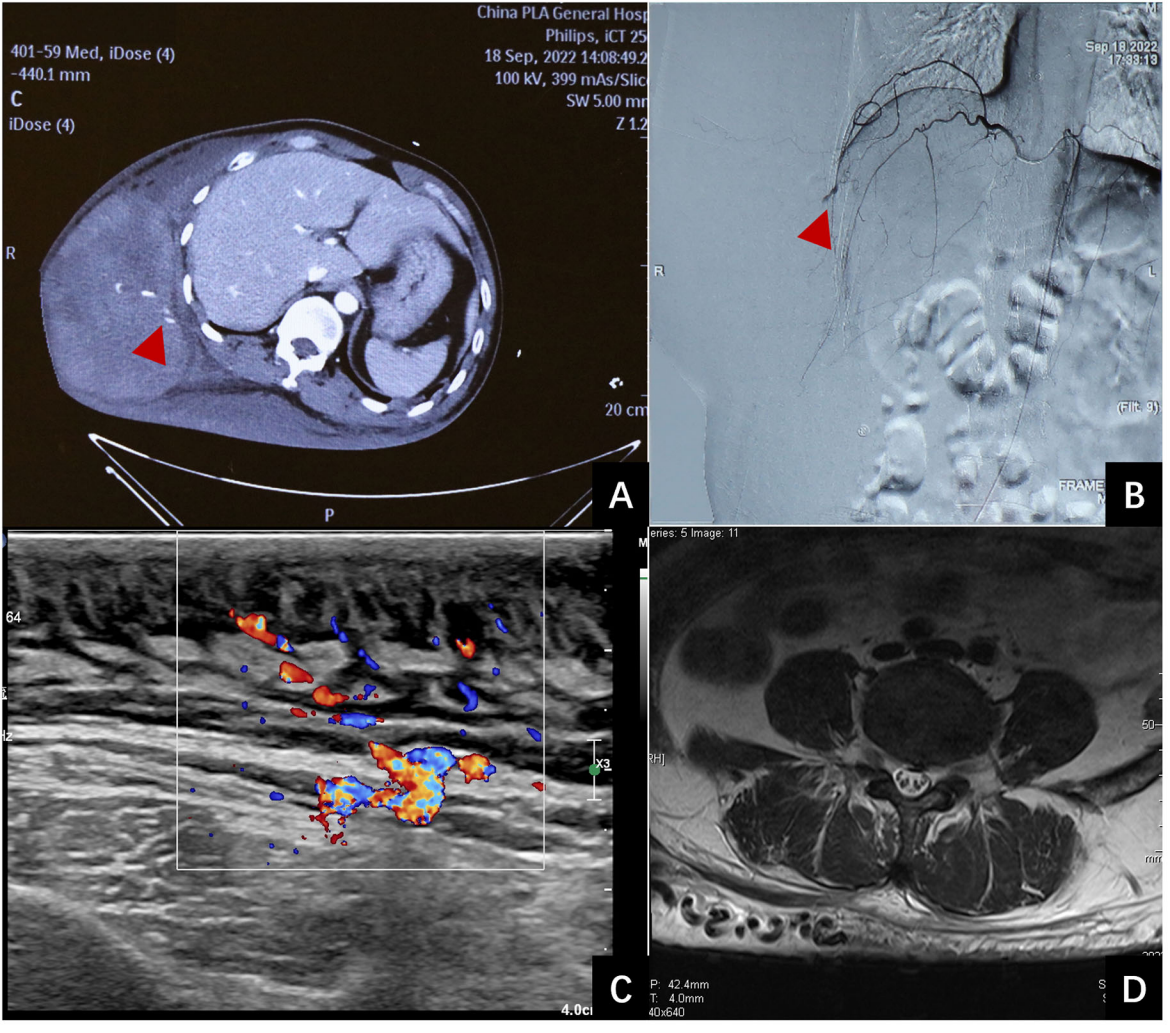
Examination of the patient. **(A)**: Contrast- enhanced computed tomography revealed extravasation of the contrast in the hematoma (red arrow). **(B)**: Emergency interventional radiology showed vascular leakage (red arrows). **(C)**: B-mode ultrasound demonstrates thickening of the skin and subcutaneous fat layer on the back, characterized by uneven echogenicity, with a maximum thickness of 2.7 cm. CDFI reveals abundant blood flow signal within the affected area. Spectral Doppler analysis exhibits arterial- and venous-like flow patterns. An increase in the thickness of the deep fascia is evident, measuring up to 2.9 mm at its thickest point. **(D)**: MRI scan reveals soft tissue thickening and enlarged vascular structures in lower back.

A 40-year-old male patient was brought to our medical center with a 10-day history of a large, painful subcutaneous hematoma on the right side of his torso following a minor trauma. Ten days ago, the patient’s right lower back was inadvertently struck by the open swinging door of a parked truck. The discomfort resulting from the blunt trauma was moderate, without any apparent skin abrasions or wounds. Two hours post-injury, the pain intensified, a pronounced lump emerged at the site of the injury, steadily growing in size. After undergoing fluid resuscitation at the local hospital, the patient was transferred to a tertiary hospital 19 hours post-injury. Upon admission, the lump on the right side of the trunk has grown to a size of 30 × 15 cm with several blisters (bullae) on its surface. The hemoglobin (HB) was 62g/L. Enhanced CT scans showed subcutaneous hematoma in the right trunk, with local contrast agent accumulation in it, indicating the presence of active bleeding (**Figure 1A**). The patient was diagnosed with a massive subcutaneous hematoma on the right side of the trunk, and hemorrhagic shock. The arteriography revealed a rupture and bleeding in the intercostal artery (**Figure 1B**), subsequent embolization was successfully executed to arrest the bleeding. Following the surgery, the patient’s HB levels remained stable and showed a gradual increase over time. During the initial three days following admission, the patient received transfusions amounting to 3150 ml of red blood cells, 1200 ml of plasma, and 20 g of human serum albumin. Subsequent to this period, no additional blood products were administered.

**Figure 2:**
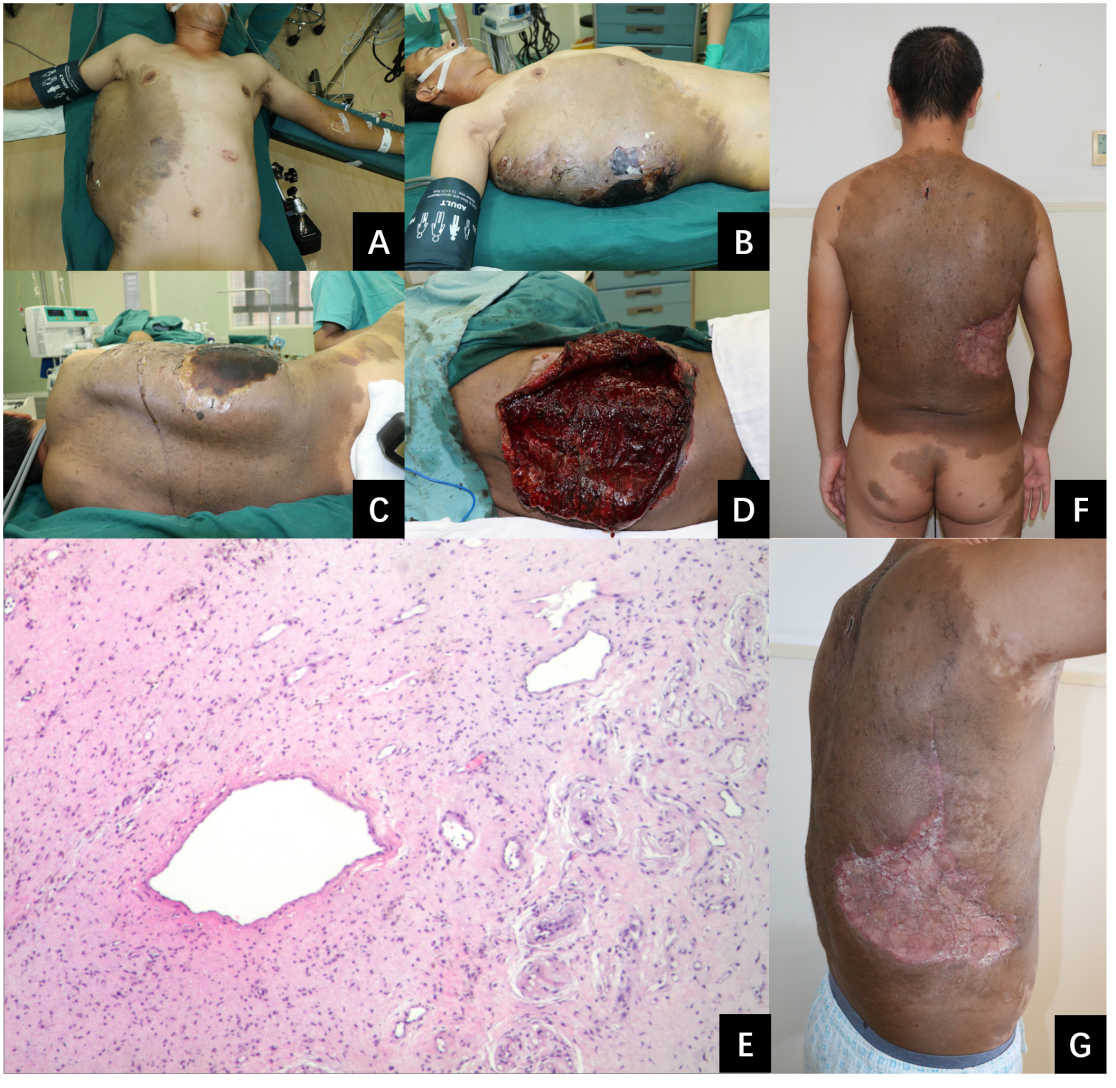
A photographic series illustrating the patient’s condition before and after our treatment. **(A–C)**: A substantial subcutaneous hematoma on the right side of the trunk. The hematoma is noted for its remarkable size, measuring 33 cm in length, 16 cm in width, and 10 cm in depth, indicating a significant accumulation of blood beneath the skin. **(D)**: The appearance of the inner wall tissue of the hematoma during the surgical procedure. It’s dark red and fragile, with a tendency to bleed upon contact. **(E)**: Pathological image. This is observed under a hematoxylin and eosin stain at an original magnification of 40 times. **(F, G)**: Successful healing of skin grafts at one month post-procedure.

**Figure 3:**
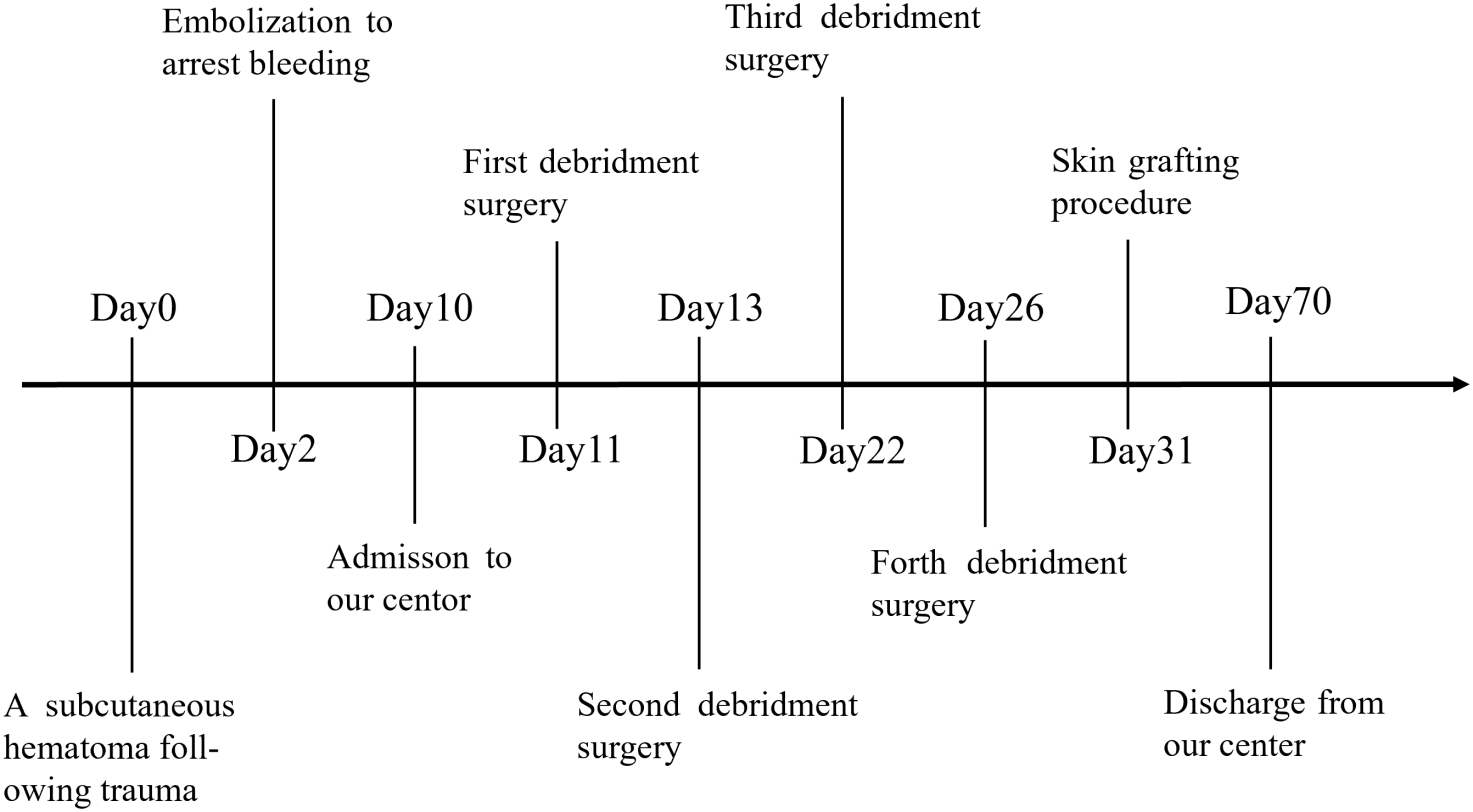
Timeline of the case.

Ten days post-injury, the patient was transferred to our medical center for management of the hematoma on the right trunk and associated necrotic tissue. His past, personal, and family history was unremarkable. After admission, physical examination revealed that the patient’s vital signs were stable. B-ultrasound (see **Figure 1C**) and MRI (see **Figure 1D**) were admitted. Multiple café-au-lait macules were observed across various regions of the body, including the trunk, buttocks, and limbs. The largest macule extended over the entire back, reaching from the spina iliaca to the neck and encompassing both armpits. It extended laterally to the left anterior axillary line, and on the right, it spanned to include areas of the right chest and abdomen. A sizable, firm mass measuring approximately 33 cm by 16 cm and with a height of 10 cm was observed on the right side of the trunk. A hardened eschar, indicative of necrotic skin, measuring approximately 20 cm by 15 cm, was present on the mass, accompanied by mild erythema and edema in the adjacent tissue (refer to **Figures 2A-C**). The entire mass was situated within the pigmented region on the trunk. The complete blood count (CBC) results indicated an elevated white blood cell (WBC) count at 15.6 × 10^9/L, decreased hemoglobin (HB) levels at 87 g/L. All additional laboratory parameters were within normal ranges. After admission, the patient received treatments including anti-infective therapy and nutritional support. On the second day following admission, an incision and drainage of the hematoma were carried out. The incision was made along the median line of the long axis of the hematoma. A total of 3000 ml of dark red blood and blood clots were evacuated. The internal surface of the hematoma displayed a dark red coloration and was fragile, readily bleeding upon contact (see **Figure 2D**). It was planned to remove the eschar and the unhealthy inner wall of the hematoma. However, throughout the resection procedure, the application of electrocoagulation and electric resection failed to provide effective hemostasis, and substantial bleeding persisted, even with the implementation of ligation techniques for blood control. Only a small portion of necrotic tissue was excised, after which the wound was closed with negative pressure wound therapy (NPWT). Two days subsequent to initial debridement, the patient persisted to have significant necrosis at the wound site, which was associated with the development of pyrexia. The complete blood count revealed a white blood cell (WBC) count of 22.4 × 10^9/L. A subsequent debridement procedure was performed to remove the eschar along with the adjacent nonviable soft tissue. In this instance, the debridement procedure was performed utilizing a technique that entailed initial suturing followed by resection. This approach required the use of 1–0 absorbable sutures to first secure the juncture between the tissue designated for removal and the tissue to be preserved. After the suturing was completed, the tissue designated for removal was meticulously excised using a scalpel or scissors, closely adjacent to the suture line. The repeated application of this method for the excision of necrotic and unhealthy tissue led to a substantial reduction in bleeding. The surgery successfully excised most of the necrotic tissue and also delicately cleared portions of the vulnerable, bleed-prone inner hematoma wall. Post-surgery, the patient’s body temperature normalized, and white blood cell counts steadily declined. Subsequently, three additional surgeries were conducted. The remaining necrotic tissue and the unhealthy inner lining of the hematoma were excised utilizing the aforementioned technique. Three weeks after the initial surgery, a skin grafting procedure using a razor-thin graft from the scalp was performed to repair the wound. Based on the pathological examination of the hematoma inner wall tissue, which showed the presence of diffuse cutaneous neurofibromas and thin-walled ectatic blood vessel (see **Figure 2E**), along with the results of the general physical examination (numerous café-au-lait macules), the patient met the diagnostic criteria for NF1 (8). The skin grafts healed well (see **Figures 2F, G**). The patient was discharged 2 months after admission. The patient was under observation for a period of twelve months and exhibited no hematoma recurrence. A timeline of the case was presented where all the important events were marked (see **Figure 3**).

## Discussion

NF1 is a complex, multisystemic, dominant genetic disorder caused by mutations on chromosome 17 (9). Angiopathy linked to Neurofibromatosis Type 1 has the potential to lead to the rupture of blood vessels and subsequent bleeding. Improper or untimely diagnosis and treatment of NF1-related angiopathy can have severe consequences, potentially leading to fatal outcomes. However, it is easy to overlook the diagnosis of NF1 in patients who experience bleeding due to minor trauma. Our patient had presented at three different health facilities prior to admission to our center, where a diagnosis of NF1 was not previously established. This oversight may be attributable to the tendency for clinicians to associate spontaneous bleeding with specific pathologies, whereas bleeding secondary to trauma is less commonly considered indicative of an underlying disorder such as NF1. And when bleeding or hematomas occur, it is the surgeons who see the patients, not the dermatologists, thus increasing the possibility of a missed diagnosis. Ten cases of NF1 patients with critical hematomas located in the trunk region have been documented (refer to **Table 1**). In the ten cases of hematoma patients, the diagnosis of NF1 had been made early before the occurrence of the hematomas, even in childhood. Our case was the only one who was not diagnosed with NF1 before coming to our center. Therefore, early diagnosis of NF1 plays a crucial role in the prevention and treatment of bleeding or hematoma.

**Table 1:** Reported cases of NF1 patients with hematomas located in the trunk region.

Number	Study	Age(yr/sex)	Area	Vector	Previous history of neurofibromatosis	Pathological examination	Hypovolemic shock	Hemostasis method	Pathology result	Outcome
1	Present study	40/M	Right chest	Minor bruise	No	33 cm × 16 cm × 10 cm	Yes	Previous embolization (intercostal arteries)	Diffuse cutaneous neurofibromas	Survival
2	Tsutsumi et al. (2012) (10)	50/M	left shoulder	Minor abrasions	Yes	25 cm in diameter	Yes	NA	NA	Survival
3	Zhang et al. (2012) (11)	20/M	Left chest	Spontaneous	Yes	25 cm × 25 cm × 20cm	Yes	Previous embolization (intercostal arteries and 1 from the first vertebral artery)	Plexiform neurofibromatosis (previously diagnosed)	Survival
4	Rao et al. (2000) (12)	25/F	Right chest	Fall from a chairlift	Yes	30 cm × 15 cm× 10 cm	Yes	No	Cutaneous neu rofibroma	Survival
5	Kaneda et al. (2011) (13)	66/F	Left chest	Spontaneous	Yes	NA	Yes	Previous embolization(left intercostal arteries (fourth to sixth) and the branch of internal thoracic artery) Spray application of fibrin glue	Neurofibroma	Dead
6	Baek et al(2007) (14)	64/F	Left back	Spontaneous	Yes	10 cm×5 cm×3 cm	Yes	NA	Diffuse neurofibroma	Survival
7	Yocum et al. (2021) (15)	29/M	Left shoulder	Spontaneous	Yes	NA	Yes	Previous embolization(left subclavian artery) Systemic tranexamic acid	Plexiform neurofibromatosis (previously diagnosed)	Survival
8	Lam et al(2022) (16)	27/M	Left gluteal	Trivial fall	Yes	20 cm × 16 cm	No	Compression	Plexiform neurofibromatosis (previously diagnosed)	Survival
9	Azhar et al(2020) (17)	21/F	Right abdominal	Spontaneous	Yes	NA	Yes	Previous embolization (right internal mammary artery and inferior epigastric artery)	Plexiform neurofibroma	Survival
10	Zhang et al(2023) (18)	52/F	Back	Slight crush	Yes	30 cm × 15 cm	No	Previous embolization	Neurofibroma with massive hemorrhage and necrosis	Survival
11	Sakaguchi et al(2023) (19)	49/M	Left abdomen	Spontaneous	Yes	NA	Yes	Embolization (left third lumbar artery)	NA	Survival

Hemostasis is essential in treating large hematomas in patients with NF1. Angiography can precisely identify the bleeding source within NF1 vascular abnormalities. Effective interventional embolization can quickly halt the bleeding and decrease the risk of hemorrhage in future surgical procedures. The rupturing of the artery and the hemorrhaging in our patient, as well as in the six previously reported cases, have been successfully halted by means of interventional embolization. However, arterial embolization does not work well in patients who were bleeding from veins and capillaries in neurofibroma tissue. The use of hemostatic materials, application of compression, and systemic administration of tranexamic acid have all demonstrated efficacy. Extensive intraoperative hemorrhage is a critical concern during neurofibroma resection procedures, due to the proliferation of fragile, ectatic vascular structures within the neoplastic tissue (20–23). By examining the pathological data (refer to **Figure 2E**), B-ultrasound imaging (see **Figure 1C**), and MRI scans (see **Figure 1D**), it’s observable that there are numerous tortuous and thin-walled ectatic blood vessels which frequently exhibit a non-functional tunica media within their structure, resulting in a diminished or absent capacity for muscular contraction. Baek et al. proposed three methods to reduce bleeding during NF1 resection: hypotensive anesthesia, preliminary sutures around the lesion, and ligation of the limited numbers of feeding vessels in the vascular malformation of the neurofibroma (14). In this case, we implemented a refined surgical approach, beginning with suturing, followed by the meticulous resection of necrotic and aberrant tissues, thereby markedly diminishing bleeding. When suturing, it’s important to apply moderate force, as the neurofibroma and the vessels contained within it are fragile. The sutures should be placed in an overlapping fashion. The necrotic tissue and neurofibroma should be carefully removed with a scalpel or scissors, trimming as close to the sutures as possible without compromising the integrity of the stitched area. Based on our experience, utilizing the aforementioned technique can significantly diminish the incidence of bleeding during the intraoperative debridement process. Following the surgical procedure, the wound was dressed using NPWT with a polyvinyl alcohol sponge. The therapy was successful, resulting in only a minimal amount of blood drainage. This suggests that NPWT is an effective option for dressing such wounds without contributing to increased postoperative hemorrhage. The extent of neurofibroma involvement in this case is considerable. Due to this, surgical efforts are concentrated on meticulously closing the wound. However, a substantial portion of the lesion remains. As a result, careful and continuous monitoring will be necessary to assess the future progression and outcomes.

## Conclusion

Minor trauma may cause severe hemorrhage in patients with NF1. A comprehensive understanding of vascular lesions of NF1 is essential. Timely diagnosis of NF1 and effective hemostatic techniques are key to successful treatment.

## Data availability statement

The original contributions presented in the study are included in the article/supplementary material. Further inquiries can be directed to the corresponding author.

## Ethics statement

Written informed consent was obtained from the individual(s) for the publication of any potentially identifiable images or data included in this article.

## Author contributions

LZ: Writing – original draft, Writing – review & editing. XW: Writing – review & editing. HY: Writing – review & editing, Funding acquisition, Supervision. WC: Writing – review & editing. MZ: Methodology, Writing – review & editing. MHZ: Conceptualization, Writing – review & editing. ZS: Investigation, Writing – review & editing. ZC: Visualization, Writing – review & editing. FZ: Formal Analysis, Writing – review & editing.
